# Adsorption and Desorption Behavior and Mechanism of Ruthenium in Nitrite–Nitric Acid System

**DOI:** 10.3390/toxics12030181

**Published:** 2024-02-27

**Authors:** Cong Li, Chao Xie, Tianjiao Jiang, Lifeng Chen, Shunyan Ning, Caiwu Luo, Qi Zheng, Ji Wang, Yuezhou Wei

**Affiliations:** 1 School of Nuclear Science and Technology, University of South China, Hengyang 421001, China; 2 School of Resources Environment and Safety Engineering, University of South China, Hengyang 421001, China; 3 Key Laboratory of Advanced Nuclear Energy Design and Safety, Ministry of Education, University of South China, Hengyang 421001, China; 4 School of Nuclear Science and Engineering, Shanghai Jiao Tong University, Shanghai 200240, China

**Keywords:** ruthenium, adsorption, high level liquid waste, nitrite–nitric acid system

## Abstract

Ruthenium is required to separate from high-level liquid waste (HLLW) because Ru is a valuable resource and is negatively influential on the vitrification process of HLLW. However, the separation of Ru is very challenging due to its complicated complexation properties. In this study, the adsorption and desorption characteristics of ruthenium on a synthesized SiPyR-N3 (weak-base anion exchange resin with pyridine functional groups) composite were investigated in nitric acid and nitrite–nitric acid systems, respectively, and the adsorption mechanism was explored. The experimental results showed that SiPyR-N3 has a significantly better adsorption effect on Ru in the nitrite–nitric acid system than in the nitric acid system, with an increase in the adsorption capacity of approximately three times. The maximum adsorption capacity of Ru is 45.6 mg/g in the nitrite–nitric acid system. The SiPyR-N3 possesses good adsorption selectivity (*SF*_Ru/other metal ions_ is around 100) in 0.1 M NO_2_^−^–0.1 M HNO_3_ solution. The adsorption processes of Ru in the two different systems are fitted with the pseudo-second-order kinetic model and Langmuir model for uptake kinetics and adsorption isotherms, respectively. The results obtained from the FT-IR, XPS, and UV absorption spectrometry indicate that NO_2_^−^ was involved in the adsorption process either as a complexing species with the metal ions or as free NO_2_^−^ from the solution. A 0.1 M HNO_3_ + 1 M thiourea mixed solution shows effective desorption performance, and the desorption efficiency can reach 92% at 328 K.

## 1. Introduction

Nuclear energy is clean, safe, and highly efficient and thus provides a promising source of energy. Therefore, it plays a key role in the development of energy strategies for carbon neutralization with the lowest hazardous emissions to the environment [1]. Considering the rapid growth of nuclear energy, it has been expected that about 3200 t of spent nuclear fuel will be produced solely in China by the year 2030 [2]. Therefore, treatment of the generated spent nuclear fuel is important. Presently, the PUREX process is found to be an effective method for the separation and recovery of uranium (U) and plutonium (Pu) from spent nuclear fuel, resulting in high-level liquid waste. However, since other fission products (FPs), such as cesium, strontium, the platinum group metals (ruthenium, palladium, and rhodium), and minor actinides (MAs), which are intensely radioactive, remain in the HLLW, further treatment of HLLW is significantly important for the minimization of radiotoxicity to the environment [3].

Ru can be in various coordination complex forms and also in a variety of oxidation states [4]. ^106^Ru with the half-life (*T*_1/2_ = 373.6 d) is one of the extremely difficult radionuclides to treat in all fission products [5]. Ru is mainly in the form of Ru(NO)^3+^ in the nitric acid solution of spent nuclear fuel, and once it is formed, it is difficult to change by substitution or oxidation–reduction reactions. In addition, Ru(NO)^3+^ easily forms more stable complexes with various ligands (such as NO_3_^−^, NO_2_^−^, etc.); hence, Ru is rather difficult to remove during the reprocessing of spent nuclear fuel. Considering the difficulty of Ru removal in a timely fashion, there are a number of drawbacks, namely, (1) in the PUREX process, part of Ru ([RuNO(NO_3_)_3_(H_2_O)_2_]) is co-extracted with U and Pu by tributyl phosphate (TBP), but it is difficult to back-extract, which negatively affects the decontamination of U and contaminates subsequent products; (2) Ru is easily oxidized into volatile RuO_4_ in the process of nuclear waste disposal, and then it causes the contamination area for RuO_4_ recondensation to expand when it comes into contact with the cold tube walls; and (3) in the vitrification process, Ru, which is insoluble in borosilicate glass, is precipitated in the molten body as RuO_2_ and a Pd-Ru-Te alloy and accumulates at the bottom of the melting furnace, which leads to an electrode short circuit and shortens the lifespan of the furnace [6]. On the other hand, Ru is an important strategic resource and an essential material in the fields of aerospace, catalysts, and electroplating [3]. However, the average abundance of Ru in the Earth’s crust is only 0.001 ppm [7]. Fortunately, it has been anticipated that 2000 tons of Ru will be present in the spent nuclear fuel used by the world by the year 2030, which is about 62% of the mineral reserves [3]. Therefore, the secondary recovery of Ru from HLLWs has the ability to optimize the PUREX process and also recycle and reuse resources, which could solve the imbalance ratio between the supply and demand of Ru resources in both scientific and industrial applications.

HLLW has extremely strong radioactivity and high acidity, and the Ru morphologies are diverse, which poses extreme challenges in the separation of Ru from nuclear waste. Currently, the widely used methods are photoreduction [8], solvent extraction [9], ion exchange [10], electrochemical techniques [11,12,13], and adsorption [14,15,16]. Among these separation methods, the advantages of the ion exchange method are high selectivity, excellent adsorption properties for rare and precious metal ions (PGMs), simple operation, and strong regenerative ability. This method tends to be more favorable among researchers in the field of radioactive nuclide removal and separation. Previously, Lee et al. [17] investigated the adsorption effects of three types of ion exchange resins (IRN 78, Dowex 1 × 8 − 400, and AR-01) on Ru and Pd from simulated radioactive waste, and it was found that these resins have better adsorption for Pd than Ru. In another study, Suzuki et al. [18] found the adsorption ability was in the order of Pd > Rh > Ru on an N,N,N-trimethylglycine functionalized styrene–divinylbenzene copolymer (AMP 03) in a 0.1 M HNO_3_ system. The traditional ion exchange materials have a good separation effect of Pd; however, the adsorption of Ru has slow kinetics and poor selectivity. Our group has been studying large pore (pore size 50–500 nm) SiO_2_-based ion exchange resins and SiO_2_-based extraction resins [19], which combine the high selectivity and good efficiency of organic extractants and organo-functional groups and the good stability of SiO_2_. These synthesized materials have been used for the separation of different radionuclides from HLLW, revealing excellent separation performance [20].

In addition, HLLW generally contains a high concentration of nitric acid (3–4 M) and a certain amount of nitrite resulting from nitric acid radiolysis and NO_3_^−^ reduction in valence adjustment. The complexes formed by [RuNO]^3+^ with NO_2_^−^ tend to be much more stable than those formed with NO_3_^−^ [21]. Therefore, the existence of nitrite has a significant influence on the adsorption efficiency of Ru [4]. However, there are currently very limited studies on the influence of nitrite on the species distribution of Ru, the use of nitrite in the auxiliary extraction of metal ions in the ion exchange system, and the in-depth exploration of the adsorption behavior and the adsorption mechanism of Ru.

Therefore, in this work, the adsorption behavior and mechanism of silica-based resins (SiPyR-N3) for Ru were investigated in nitrite–nitric acid systems. The sorption properties of Ru(III) ions in different nitric acid and nitrite concentrations were explored. The sorption properties were examined with different reaction times, initial metal ion concentrations, sorption temperatures, and coexisting ions (to investigate the selectivity of multicomponent solutions) to determine their effects on adsorption performance. In addition, the adsorption mechanism of SiPyR-N3 towards Ru was also explored. After that, the elution behavior of adsorbed Ru was investigated using different reagents.

## 2. Materials and Methods

### 2.1. Reagents

Porous SiO_2_ (average diameter of 75–150 μm) was purchased from Fuji Silysia Chemical Ltd., Kasugai, Japan. Ru(NO)(NO_3_)_x_(OH)_y_ (x + y = 3, analytical grade, 1.5 wt.% Ru), 4-vinylpyridine (4-VP, purity 95%), divinylbenzene (DVB, purity 55%), and nitrile-based cyclohexane (V-40, purity 98%) were purchased from Macklin Biochemical Technology Co., Ltd., Shanghai, China (www.macklin.cn, accessed on 20 February 2024). All the other reagents, such as nitric acid, hydrochloric acid, methanol, diethyl phthalate (DEP), azobisisobutyronitrile (AIBN), and acetophenone (ACP), were analytical grade and purchased from Sinopharm Chemical Reagent Co., Ltd., Shanghai, China (www.reagent.com.cn, accessed on 20 February 2024). All the solutions in the present experiment were prepared with ultrapure water.

### 2.2. Synthesis of Materials

**Figure 1:**
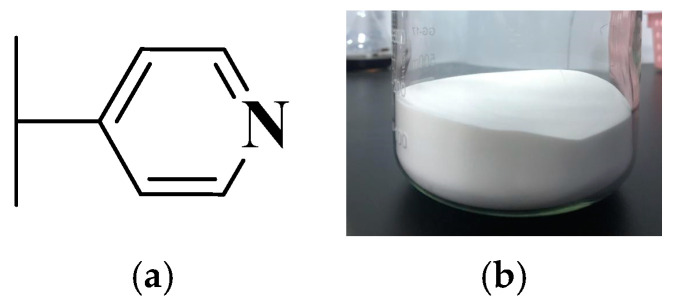
(**a**) Skeletal structure of SiPyR-N3 and (**b**) photograph of SiPyR-N3 powder.

SiPyR-N3 was described as a kind of pyridine-type weakly basic anion exchange resin obtained through a one-pot copolymerization reaction using 4-vinylpyridine (as a monomer) and divinylbenzene (as a crosslinking agent). The preparation of this material was previously reported in our work [22]. In detail, 6.41 mL of DVB, 17.69 mL of 4-VP, 45 mL of ACP, 30 mL of DEP, 0.3215 g of AIBN, and 0.2144 g of V-40 were mixed thoroughly. Then, the mixed solution was suctioned into the rotary evaporator using a reverse suction method under vacuum conditions and was put in full contact with 100 g of SiO_2_. The oil phase gradually entered the pores of the SiO_2_ substrate through capillary action. After sufficient mixing, nitrogen gas was injected to restore the inside of the flask to normal pressure, and the temperature was gradually changed for heating [23]. The chemical structure and photograph (of the powder form) of SiPyR-N3 are shown in Figure 1a,b, respectively.

**Figure 2:**
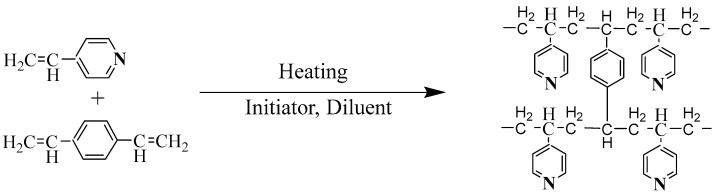
SiPyR-N3 synthesis route.

The synthesis route is shown in Figure 2 [23].

### 2.3. Adsorption Experiments

Batch adsorption was used to evaluate the adsorption and desorption performance of the prepared SiPyR-N3 for Ru ions. The experiment was conducted in a glass bottle with a screw cap at 0.02 g mL^−1^ solid–liquid ratio, followed by mechanical oscillation at a constant speed of 160 revolutions per minute (RPM). After solid–liquid separation using a filter membrane (1.2 mesh), the concentrations of Ru and other metal ions were determined by an inductively coupled plasma spectrometer (ICP-AES). The adsorption capacity *Q* (mg·g^−1^), adsorption efficiency *E* (%), distribution coefficient *K_d_* (mL g^−1^), and separation factor *SF_A_*_/_*_B_* toward Ru were calculated, as shown in Equations (1)–(4).

(1) $$Q=\frac{\left(C_{0}-C\right)}{m}\times V,$$

(2) $$E=\frac{\left(C_{0}-C\right)}{C_{0}}\times 100\%,$$

(3) $$K_{d}=\frac{\left(C_{0}-C\right)}{C}\times \frac{V}{m},$$

(4) $${{SF}_{A/B}=K}_{dA}/K_{dB},$$

where *C*_0_ and *C* represent the concentration of Ru in the solution before and after adsorption, respectively. Furthermore, *V* and *m* represent the volume of the Ru initial solution and the mass of the adsorbent, respectively.

### 2.4. Desorption Procedure

Desorption should also be considered in practical applications, as it is considered an important parameter for the evaluation of adsorbent performance. The effects of desorption agents, desorption time, and temperature on the desorption rate were studied. SiPyR-N3 after static adsorption (*V*/*m*: 5 mL/0.1 g, [metal ions]: 2 mmol L^−1^, time: 24 h, temperature: 298 K, [HNO_3_]: 0.1 mol L^−1^, [NaNO_2_]: 0.1 mol L^−1^) was washed with ultrapure water until the filtrate pH was constant and separated using a Brinell funnel; then, it was put into a vacuum dryer, before being used for subsequent desorption experiments. The static desorption experiment was conducted in a constant-temperature water bath. The desorption performance was evaluated by calculating the desorption quantity *Q_d_* (see Equation (5)) and the desorption efficiency *E_d_* (see Equation (6)) based on the measured Ru concentration.

(5) $${Q_{d}=C}_{d}\times \frac{V}{m},$$

(6) $$E_{d}=\frac{Q_{d}}{Q}\times 100\%,$$

where *Q_d_* and *Q* (mg·g^−1^) are the desorption quantity and the adsorption capacity of Ru by a gram of resin, and *C_d_* represents the concentration of Ru in the solution after elution. *V* and *m*, respectively, represent the volume of the eluting agent and the mass of SiPyR-N3 after adsorption.

### 2.5. Adsorption Mechanism Study

In order to study the type of binding on the SiPyR-N3 surface and the subsequent adsorption mechanism, FT-IR (IRTracer-100, SHIMADZU; Kyoto, Japan) was employed to detect the functional groups of the material after treatment with nitric solution and the nitrite and nitric mixture. In detail, these samples were firstly dried at 60 °C before being ground with KBr (at a mass ratio of 100:1: *w*/*w*) and then pressed into a KBr disc. Furthermore, UV spectroscopy (UV-600 Plus, SHIMADZU; Kyoto, Japan) was applied to analyze Ru species in the NaNO_2_-HNO_3_ solution. The absorption spectra of the metal ions in different concentrations of NaNO_2_ solutions were measured in the wavelength range of 300 to 450 nm. In detail, a mixture of different concentrations of NaNO_2_ and ruthenium was diluted 10 times, and a corresponding concentration of NaNO_2_ solution was selected for the blank sample to determine the mixed solution absorbance. SEM (Hitachi Regulus8100, HITACHI; Tokyo, Japan) was used for studying the morphological structure of the surface. The chemical composition and the elemental contents were detected using EDS (Ultima Expert, Kyoto, Japan). XPS (Thermo ESCALAB 250 XI, THERMO FISHER; New York, NY, USA) analysis was used. The survey spectra were obtained for the global identification of the main elements, in which the core levels (signal deconvolution) were adjusted after calibration of the binding energy (BE, eV).

## 3. Results and Discussion

### 3.1. Evaluation of Adsorption Performance

#### 3.1.1. Effects of HNO_3_, NaNO_2_, and NaNO_3_ Concentration

**Figure 3:**
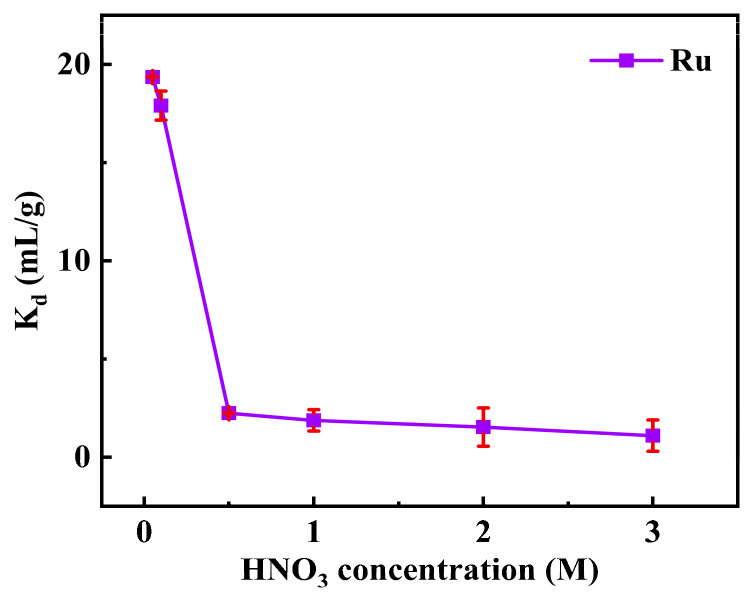
Effect of initial HNO_3_ concentration on adsorption of Ru (*m/V* = 0.02 g mL^−1^, *C*_Ru_ = 2 mmol L^−1^, *T* = 298 K).

Figure 3 shows the effect of nitric acid concentration on the *K_d_* of Ru. The adsorption results of Ru by SiPyR-N3 with weak base groups showed that the *K_d_* decreased significantly with the increase in HNO_3_ concentration, and the *K_d_* of Ru dropped from 19.4 mL/g to 1.1 mL/g in the range of 0.1~3 M HNO_3_. The reason may be that a high H^+^ concentration inhibits the deprotonation and ion exchange capacity of the adsorbent, which enhances the repulsion force with the protonated quaternary amine groups on the resins and decreases the adsorption with Ru species (Figure S1), so the adsorption decreased with nitric acid concentration [22].

**Figure 4:**
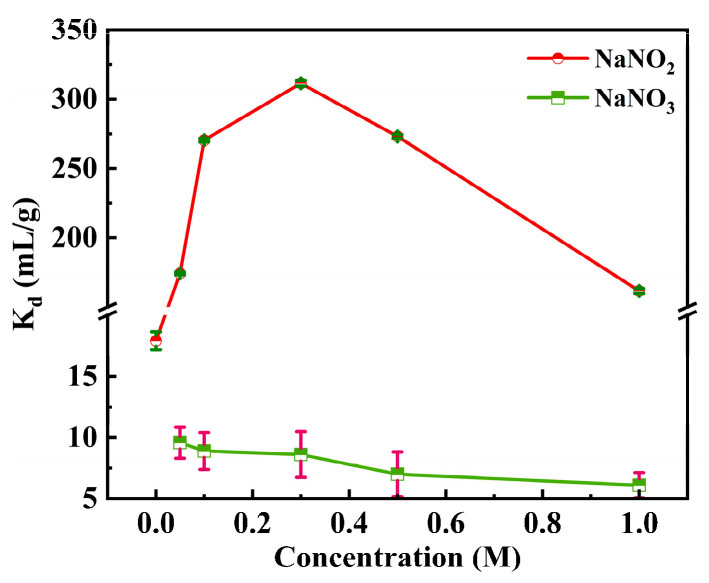
Effect of initial NaNO_2_ and NaNO_3_ concentration on adsorption (*m/V* = 0.02 g mL^−1^, *C*_Ru_ = 2 mmol L^−1^, *C*_HNO_3__ = 0.1 mol L^−1^, *T* = 298 K).

Figure 4 shows the effect of the NaNO_2_ and NaNO_3_ concentration on the adsorption of Ru. Interestingly, the *K_d_* and the adsorption capacity increased significantly with the increase in NaNO_2_ concentration (Figure S2). The maximum *K_d_* reaches 311 mL/g in 0.1 M HNO_3_-0.3 M NO_2_^−^. It is possible that different concentrations of NO_2_^−^ and NO_3_^−^ coordinate with [RuNO]^3+^ to form a variety of Ru nitrite complexes, such as RuNO(NO_2_)(NO_3_)_2_(H_2_O)_2_, etc. [24]. When the concentration of NaNO_2_ increased above 0.3 M, the adsorption capacity slightly decreased, which may be due to the competitive adsorption between the excess NO_2_^−^ and ruthenium complexes. For 0.1 M NaNO_2_, the *K_d_* reached 270 mL/g. Because of the suitable difference in the *K_d_* values in 0.1 M and 0.3 M NaNO_2_ solutions and the slight differences in the adsorption efficiency and adsorption capacity (Figure S1), additionally, the redox reaction was performed between nitric acid (strong oxidizing agent) and sodium nitrite, a more stable 0.1 M NaNO_2_ system was selected for subsequent adsorption experiments. In addition, the effect of the NaNO_3_ concentration on the adsorption of Ru was also studied. As shown in Figure 4, the adsorption effect gradually deteriorates as the concentration of NO_3_^−^ increases. In nitric acid solution, Ru forms nitrate–water complexes (Ru(NO)(NO_3_)_x_(H_2_O)_5-x_]^3-x^). With the increase in the nitric acid concentration, the chemical species (Ru(NO)(NO_3_)_3_(H_2_O)_2_, [Ru(NO)(NO_3_)_4_H_2_O]^−^, Ru(NO)(NO_3_)_5_]^2−^, and [Ru(NO_3_)_6_]^4−^) of Ru change as follows:

$${\left[Ru(NO){\left({NO}_{3}\right)}_{x}{\left(H_{2}O\right)}_{5-x}\right]}^{3-x}+{{NO}_{3}}^{-}\to {\left[Ru(NO){\left({NO}_{3}\right)}_{x+1}{\left(H_{2}O\right)}_{4-x}\right]}^{2-x}+H_{2}O.$$

Therefore, as the concentration of nitrate increases, more active sites are needed for ion exchange and adsorption with Ru(NO)^3+^. According to the different adsorption properties in NO_2_^−^ and NO_3_^−^ systems, it is deduced that the Ru species formed in the NO_2_^−^ solution are more stable and easily adsorbed by SiPyR-N3 [25].

#### 3.1.2. Kinetics Studies

**Figure 5:**
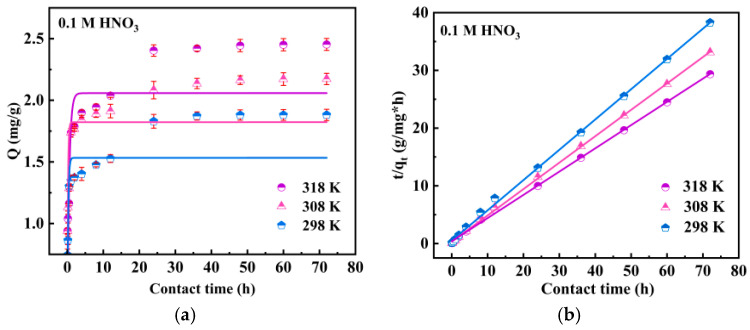

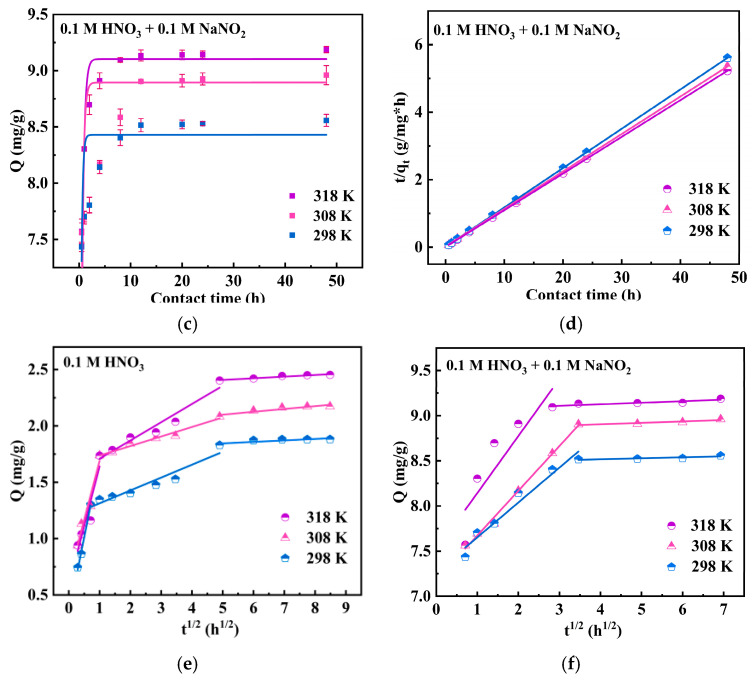
Pseudo-first-order kinetic fitting (**a**) and pseudo-second-order kinetic fitting (**b**) of Ru in nitric acid system. Pseudo-first-order kinetic fitting (**c**) and pseudo-second-order kinetic fitting (**d**) of Ru in the nitrite–nitric acid system. Internal particle diffusion model in nitric acid system (**e**) and in the nitrite–nitric acid system (**f**). (*m/V* = 0.02 g mL^−1^, *C*_Ru_ = 2 mmol L^−1^, *C*_HNO_3__ = 0.1 mol L^−1^, *C*_NaNO_2__ = 0.1 mol L^−1^).

The adsorption kinetics of Ru on SiPyR-N3 in 0.1 M HNO_3_ solution and 0.1 M HNO_3_-0.1 M NaNO_2_ solution at different temperatures were studied, respectively. Firstly, it is observed from Figure 5a that the adsorption equilibrium was reached at 24 h in the 0.1 M HNO_3_ solution. As the temperature increases, the adsorption capacity slightly increases, indicating that the adsorption process is endothermic. Figure 5c shows the effects of temperature and time on the adsorption of Ru in the nitrite–nitric acid system. The adsorption equilibrium was reached after 12 h, which is nearly one times faster than that in the nitric acid system. Furthermore, the adsorption capacity at 298 K was 8.5 mg/g, which is four times more than that in NO_3_^−^, and it increased slightly with the increase in temperature. Comparing these results with those obtained from 0.1 M HNO_3_ on Ru adsorption by SiPyR-N3, the adsorption rate and adsorption capacity were significantly increased, and the *K_d_* of Ru increased by 15 times in 0.1 M NaNO_2_-0.1 M HNO_3_. The experimental results show that the addition of NaNO_2_ is beneficial to the adsorption of Ru on SiPyR-N3. As the temperature increased from 298 K to 318 K, the adsorption capacity of SiPyR-N3 for Ru increased from 8.57 mg/g to 9.18 mg/g, indicating that the reaction is an endothermic process.

The adsorption kinetics were further analyzed by the pseudo-first-order kinetics equation (PFORE, Equation (7)) and pseudo-second-order kinetics equation (PSORE, Equation (8)):

*ln(Q_e_ − Q_t_) = ln Q_e_ − k*_1_*t,* (7)

(8) $$\frac{t}{Q_{t}}=\frac{1}{k_{2}Q_{e}^{2}}+\frac{t}{Q_{e}},$$

where *Q_t_* is the adsorption capacity at *t* (h), *Q_e_* (mg g^−1^) is the adsorption capacity at equilibrium, and *k*_1_ (h^−1^) and *k*_2_ (mg g^−1^ h^−1^) are the constants of the PFORE and PSORE, respectively.

The fitting results are shown in Figure 5 and Table S1. In both systems, the PSORE showed a better fitting of the experimental data than the PFORE, and its correlation coefficient (*R*^2^) was closer to 1. In addition, the calculated *Q_e_* value of the pseudo-second-order kinetics model had excellent consistency with the actual adsorption equilibrium capacity (*Q*_*e*,*exp*_). Because the pseudo-second-order model is more suitable for describing the ruthenium adsorption process, it is indicated that the adsorption reaction of Ru on SiPyR-N3 is chemisorption.

In order to further determine the control steps of the adsorption rate, the intraparticle diffusion equation was used for fitting (Equation (9)). The transfer of adsorbed substances during solid–liquid adsorption can be characterized as boundary layer diffusion or intraparticle diffusion.

(9) $${Q_{t}=k}_{i}t^{1/2}+C,$$

where *Q_t_* (mg g^−1^) is the adsorption capacity at time *t* (h), *k_i_* is the intraparticle diffusion constant, and *C* is an arbitrary constant that represents the thickness (or resistance) of the boundary layer.

In particular, the intraparticle diffusion model was fitted for both systems (Figure 5e,f). The trilinear and bilinear regions were investigated for the nitric acid and the nitrite–nitric acid systems, respectively, indicating that both belong to a multistep adsorption mechanism throughout the entire process. In both systems, the slope of step 1 is greater than that of step 2. As the adsorption reaction proceeded, the following steps tended to the adsorption equilibrium, indicating it was not a restricting step for Ru adsorption. Moreover, the fitting curves of both systems do not pass through the origin, inferring that the adsorption includes intradiffusion and chemisorption, and its rate is controlled by chemisorption. The differences in the linear region between the two may be due to the addition of nitrite. The slope of step 1 increased with the increase in temperature, indicating that the SiPyR-N3 material has a faster reaction rate for Ru adsorption at higher temperatures.

#### 3.1.3. Isotherm Studies

**Figure 6:**
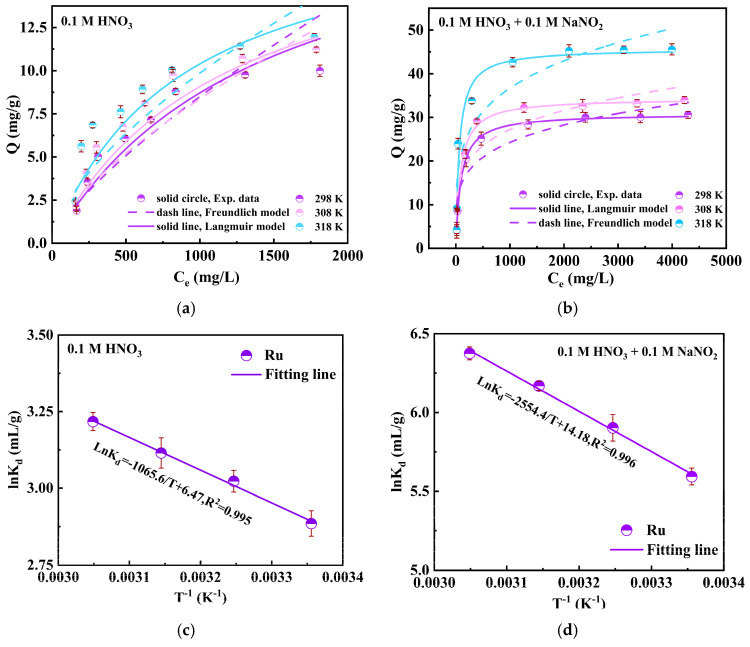
Adsorption isotherms of Ru in 0.1 M HNO_3_ solution (**a**), adsorption isotherms of Ru in 0.1 M HNO_3_ + 0.1 M NaNO_2_ solution (**b**). Relationship diagram between *lnK_d_* and *T*^−1^ of Ru in nitric acid system (**c**) and in the nitrite–nitric acid system (**d**). (*m/V* = 0.02 g mL^−1^, *C*_HNO_3__ = 0.1 mol L^−1^, *C*_NaNO_2__ = 0.1 mol L^−1^).

Figure 6a,b show the relationship between the adsorption capacity and the initial concentration of Ru in the nitric acid system and the nitrite–nitric acid system, respectively. Firstly, in the HNO_3_ solution, the adsorption capacity of SiPyR-N3 increased as the concentration of Ru increased before it reached saturation, and it increased with the temperature but just from 9.8 mg/g to 11.9 mg/g, which indicated that the adsorption capacity of materials for Ru is limited in a nitric acid system and the increase in temperature is marginally helpful for adsorption. Although the adsorption capacity is increased by 21% with the temperature, it is still too small and cannot reach the desired efficiency. Secondly, in the HNO_3_-NO_2_^−^ solution, the adsorption capacity of SiPyR-N3 for Ru also follows the pattern of a steep increase, then saturation, and then stabilization. The maximum adsorption capacity of SiPyR-N3 for Ru was 30.5 mg/g at 298 K in the HNO_3_-NO_2_^−^ system, almost three times more than that in the HNO_3_ system. It indicated that nitrite enhances the adsorption affinity. Furthermore, the saturated adsorption capacity significantly increased from 30.5 mg/g to 45.6 mg/g with the increase in temperature from 298 K to 318 K. Two isotherm models are used to fit the experimental data, namely, the Langmuir isotherm model (associated with monolayer adsorption, without any interactions of the adsorbed molecules; homogeneous adsorption) (Equation (10)) and the Freundlich isotherm model (multilayer sorption with possible interactions of the adsorbed molecules; heterogeneous distribution of the adsorption energies) (Equation (11)).

(10) $$Q_{e}=\frac{q_{m}\times K_{L}\times C_{e}}{1+K_{L}\times C_{e}},$$

(11) $$Q_{e}=K_{F}\times {C_{e}}^{\frac{1}{n}},$$

where *Q_e_* (mg g^−1^) is the equilibrium uptake amount, and *q_m_* (mg g^−1^) and *C_e_* (mg L^−1^) are the calculated saturation adsorption capacity and equilibrium ion concentration in solution, respectively. *K_L_* (L mg^−1^) is the Langmuir constant, and *K_F_* (mg^1−n^ L^n^ g^−1^) and *n* are the Freundlich constants.

The fitting parameters are shown in Table S2. The correlation coefficients (*R*^2^) of the Langmuir isotherm mode under both systems are higher than 0.95; meanwhile, they are also exposed to different temperatures. Obviously, the Langmuir model had a higher fitting coefficient than the Freundlich model in both systems. Therefore, the Langmuir model exhibits a better description of the Ru adsorption process. The fitted *Q_m_* values are very close to the experimental (*Q*_*e*,*exp*_) values (with errors of less than 5%). Hence, it can be speculated that the adsorption process of Ru on SiPyR-N3 is mainly monolayer chemisorption.

#### 3.1.4. Adsorption Thermodynamics

The adsorption thermodynamics are shown in Figure 6c,d and Table S3. Based on kinetic and isotherm results, the adsorption capacity increased with increasing temperature. The main thermodynamic parameters can be obtained based on the van ’t Hoff equation [26,27,28,29]:

(12) $$\mathrm{Ln}K_{d}=-\frac{\Delta H{}^{\circ}}{RT}+\frac{\Delta S{}^{\circ}}{R},$$

(13) ΔG°=ΔH°−ΔS°T,

where ΔG° (J mol^−1^), ΔH° (J mol^−1^), and ΔS° (J K^−1^ mol^−1^) are the changes in the Gibbs free energy, enthalpy, and entropy, respectively. *R* (8.314 J K^−1^ mol^−1^) represents the gas constant at temperature *T* (K).

As shown in Table S3, the values of ΔH° and ΔS° are positive, while ΔG° is negative, which indicates that the adsorption process is endothermic. This is consistent with the observation results of the experiment. The negative value of ΔG° indicates that adsorption is spontaneous. A positive ΔS° value indicates that the adsorption process is entropy-driven. The differences in parameters in both systems are mainly due to the diverse Ru complexes formed, which show different stability and affinity.

#### 3.1.5. Adsorption Selectivity Experiment

**Figure 7:**
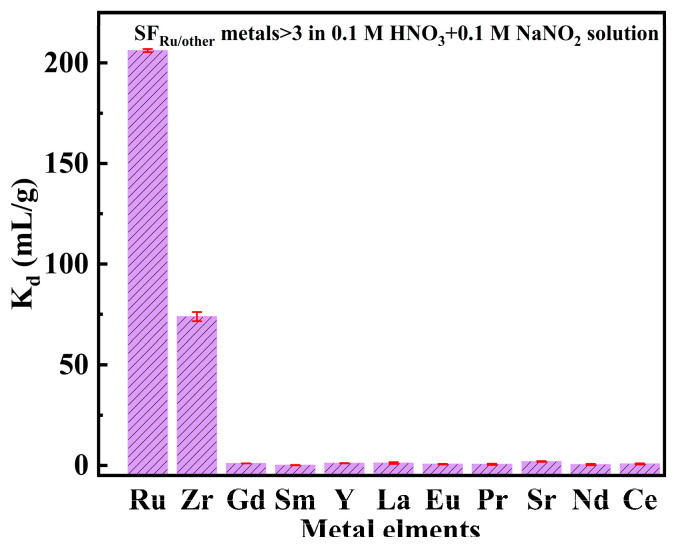
Distribution coefficients of typical metal elements in HLLW. (*m/V* = 0.02 g mL^−1^, *C*_[metal ion]_ = 2 mmol L^−1^, *C*_HNO_3__ = 0.1 mol L^−1^, *C*_NaNO_2__ = 0.1 mol L^−1^, *T* = 298 K).

The HLLW is characterized as very complex with multiple metal ions, such as Sm, Y, Ce, Pr, Gd, La, Eu, Nd, Zr, Sr, etc., except Ru. Therefore, in order to evaluate the selectivity of SiPyR-N3 for Ru in natural samples, the adsorption experiments on simulated HLLW were carried out in the HNO_3_-NO_2_^−^ system. As shown in Figure 7, SiPyR-N3 exhibited poor adsorption of the coexisting metal ions except Zr. The *K_d_* of Ru was as high as 207 g·mL^−1^ in the multiple metal ion mixed solution and was obviously higher than other metal ions (*K_d_*_(Zr)_ = 72 g·mL^−1^, *K_d_*_(other metals)_ < 1.2 g·mL^−1^). Furthermore, the *SF*_Ru/other metal ions_ is greater than 99.8, except for *SF*_Ru/Zr_, indicating that SiPyR-N3 has excellent adsorption performance and good selectivity for Ru. Partial Zr was adsorbed by SiPyR-N3 due to the strong affinity between Zr and N−containing groups [30]. Regarding Pd, which is also a platinum group metal, previous articles have reported relevant research [22]. Generally, SiPyR-N3 has good adsorption ability and selectivity for Ru in HLLW.

### 3.2. Interaction Mechanism Study

#### 3.2.1. FT-IR Analysis

**Figure 8:**
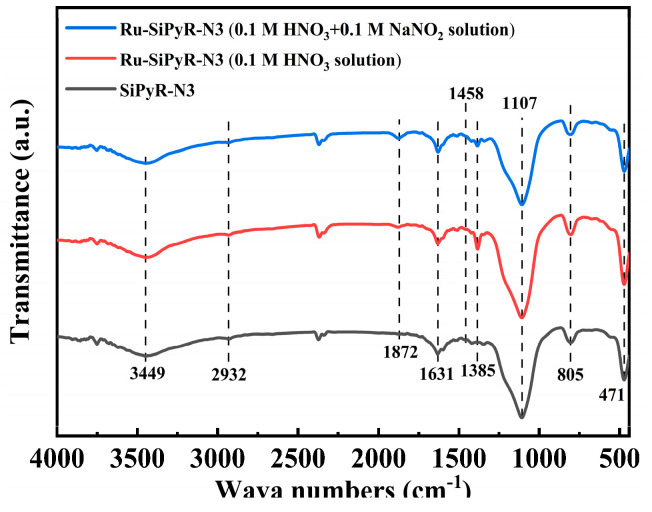
FT-IR spectra of fresh and Ru-loaded SiPyR-N3.

To confirm successive grafting and emphasize the changes that occurred in the functional groups before and after adsorption, SiPyR-N3 was characterized by FT-IR. As shown in Figure 8, the Si-O appeared with three identical peaks at around 1107 cm^−1^ (Si-O-Si bands overlapped with δ_CH_ (out-of-plane) [20]), 805 cm^−1^ (Si-O-Si bands, and the increase in the intensity after adsorption is mainly due to overlapping with NO_3_^−^ from the solution), and 471 cm^−1^ (Si-O bands). The clear characteristic peak at 1385 cm^−1^ after adsorption of Ru by SiPyR-N3 is in connection with NO_3_^−^, indicating the involvement of NO_3_^−^ in the adsorption process. The absorption peaks at 1458 cm^−1^ and 1631 cm^−1^ belong to the characteristic peaks of the pyridine ring with Si-OH for SiO_2_, and there is almost no change after adsorption. Moreover, a new absorption peak appeared at the position of 1872 cm^−1^ after adsorption. The weak peak at 2932 cm^−1^ belongs to alkyl C-H. The peak at 3449 cm^−1^ comes from the O-H bond of adsorbed H_2_O.

#### 3.2.2. UV Absorption Spectra Analysis

**Figure 9:**
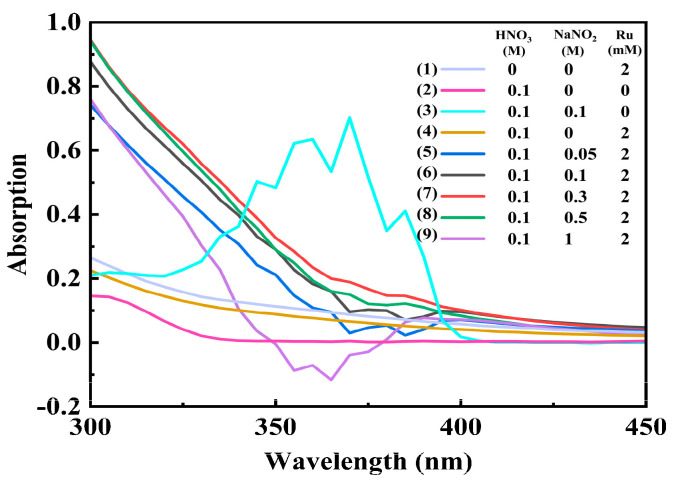
UV absorption spectrum of Ru in sodium nitrite solution.

Figure 9 shows the UV absorption spectra of Ru in nitrite–nitric acid solution. Firstly, there are multiple obvious absorption peaks at 325~400 nm only in the case of the presence of nitrite [31]. In contrast, the spectral curve is smooth, and there are no absorption peaks without nitrite in the solution. In detail, in Figure 9 curves (1) to (3), nitrite has strong absorption peaks at 325~400 nm, while Ru has no absorption peak. In the 0.1 M NO_2_^−^-0.1 M HNO_3_ system (in Figure 9 curves (3) and (6)), the peaks between 325 and 400 nm decreased significantly after adding Ru to the solution, which indirectly provides evidence of the formation of complexes between Ru and NO_2_^−^ and exhibits the complexing ability of nitrite and ruthenium at different concentrations. In addition, although the intensity of these peaks decreased obviously, weak peaks between 325 and 400 nm with a certain regularity can still be observed. In detail, in Figure 9 curves (4) to (9), the UV spectral absorption peaks are ranked from strong to weak in the order (7) > (8) > (6) > (5) > (1), where the corresponding NaNO_2_ concentration is 0.3, 0.5, 0.1, 0.05, and 1 M, respectively, which is consistent with the results of the batch adsorption experiments (Figure 4). In light of the above results, it can be speculated that the concentration of nitrite in solution decreases due to the formation of Ru–nitrite complexes ([RuNO(NO_3_)(NO_2_)_2_(H_2_O)_2_]^0^, [RuNO(NO_2_)_2_(H_2_O)_3_]^+^, etc.), and the adsorption is better with the higher peaks in this work [25]. The degree of Ru complexation varies with the concentration of nitrite added, thereby affecting the adsorption effect of SiPyR-N3 on Ru.

#### 3.2.3. SEM-EDS Analysis

**Figure 10:**
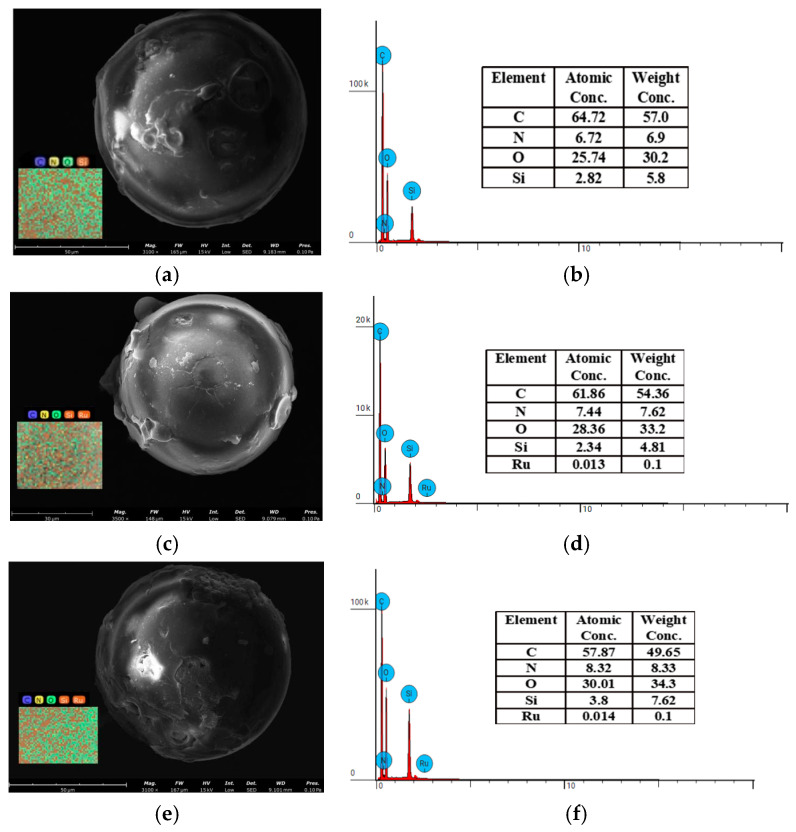
SEM-EDS characterization of SiPyR-N3 material (**a**,**b**) after adsorption in nitric acid system (**c**,**d**) and in the nitrite–nitric acid system (**e**,**f**).

Figure 10a,c,e show the SEM images of SiPyR-N3 before and after Ru adsorption. The adsorbent maintained the original size and morphological characteristics after adsorption, indicating that the material had good mechanical stability. Based on the elemental maps and the atomic proportions of C, N, O, Si, and Ru, it is further confirmed that Ru is successfully adsorbed. The amount of N and O increased significantly after adsorption, which may be due to the formation of Ru and nitrite complexes.

#### 3.2.4. XPS Analysis

**Figure 11:**
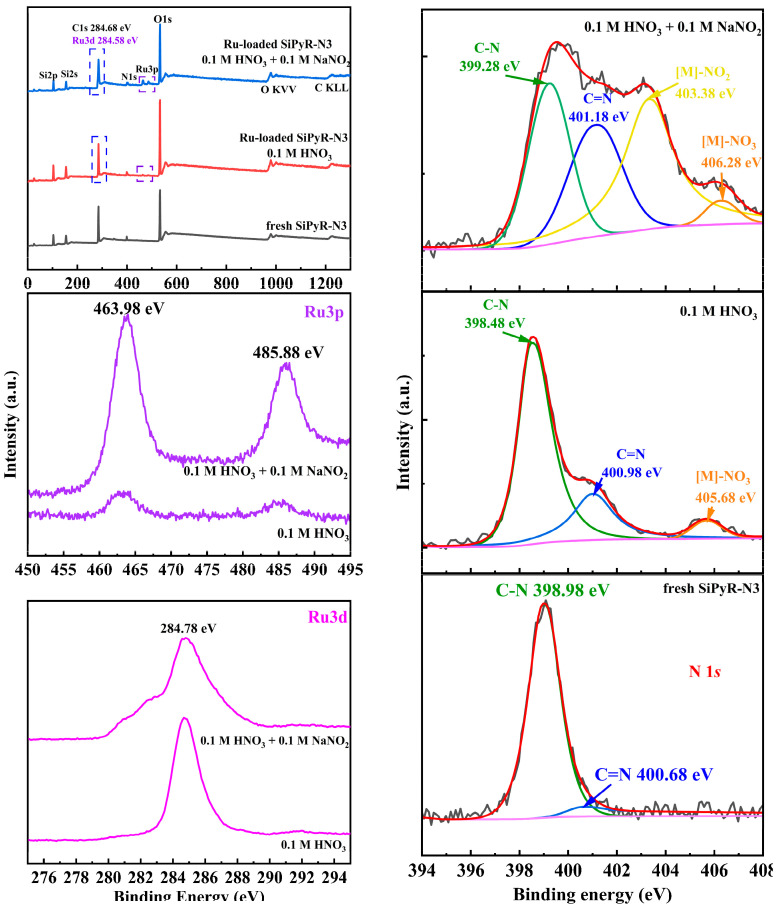
XPS survey scans of fresh SiPyR-N3 and Ru-loaded SiPyR-N3.

In order to further investigate the Ru adsorption mechanism by SiPyR-N3, XPS analysis was used to identify the changes in chemical bonds of SiPyR-N3 after adsorption, as shown in Figure 11. The silica core is confirmed by signal Si 2p and Si 2s, while the organic support is identical through the presence of C 1s, O 1s, and N 1s. The presence of Ru is confirmed by signals Ru 3d and Ru 3p, which emphasize the successful adsorption of Ru. It was observed that the peak area of Ru 3*p* is bigger in the 0.1 M HNO_3_-0.1 M NaNO_2_ system than in the 0.1 M HNO_3_ system, indicating a better adsorption performance. In addition, the change in N peak before and after adsorption was observed clearly. In detail, the N 1*s* spectrum of the pristine adsorbent SiPyR-N3 has two deconvoluting peaks at 398.98 and 400.68 eV, belonging to C–N and the tertiary amine on the pyridyl ring, respectively. In the HNO_3_ system, these peaks shifted from 398.98 and 400.68 eV to 398.48 and 400.98 eV, respectively, after the adsorption of Ru, speculating that the complexation of pyridine–N with Ru affects the electronic chemical environment of N 1*s*. The new N 1*s* peaks at 405.68 eV are contributed by Ru–NO_3_^−^. In the NaNO_2_-HNO_3_ system, the peaks of C–N, C=N, and Ru-NO_3_^−^ shifted after adsorption; furthermore, a new peak at 403.38 eV corresponds to M (Ru, Na)–NO_2_, proving the complexations of Ru and nitrite are adsorbed on the SiPyR-N3 [32,33,34,35,36,37].

### 3.3. Desorption Behavior

**Figure 12:**
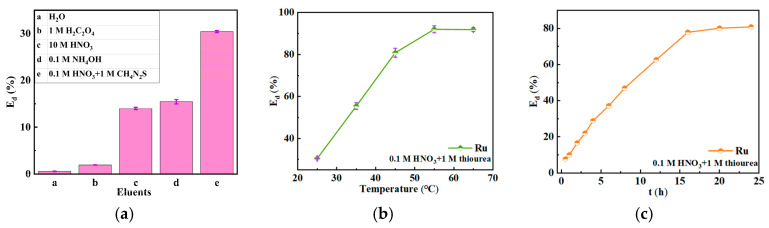
Desorption efficiency of ruthenium by different desorption agents at 298 K (**a**). Effect of temperature on desorption efficiency (**b**). Kinetic study on desorption process at 318 K (**c**). (Adsorption conditions: Ru = 2 mmol L^−1^, medium = 0.1 M HNO_3_-0.1 M NaNO_2_, *m/V* = 0.02 g mL^−1^).

Figure 12a shows the desorption efficiency of SiPyR-N3 loaded with Ru using different desorption agents. Five typical desorption agents were selected, including H_2_O, 1 M oxalate, 10 M HNO_3_, 0.1 M NH_4_OH, and 0.1 M HNO_3_ + 1 M thiourea. H_2_O has almost no desorption effect on the adsorbed Ru, and the desorption efficiency of oxalate is also very weak. The desorption effect of HNO_3_ and NH_4_OH on Ru is not significantly different, with desorption efficiency of 14% and 15%, respectively. The 0.1 M HNO_3_-1 M CH_4_N_2_S solution has the best desorption ability but only with the *E_d_* of 30% at 298 K. The desorption results prove that Ru is mainly adsorbed by chemisorption in the NaNO_2_-HNO_3_ solution and is difficult to desorb. Further, the effect of temperature on the desorption efficiency was explored, as shown in Figure 12b. The increase in temperature could contribute much to improving the desorption efficiency, and the efficiency was basically unchanged above 328 K, so 328 K is the optimal desorption temperature. Since the *E_d_* of 318 K reached 81%, the desorption kinetics of Ru were investigated at this temperature. As shown in Figure 12c, the desorption approaches equilibrium at 16 h.

## 4. Conclusions

In this work, the adsorption and desorption behavior of Ru(III) with SiPyR-N3 adsorbent was systematically investigated in nitric acid and nitrite–nitric acid systems, respectively. The distribution coefficient of Ru was assigned to 270 mL/g in the 0.1 M NaNO_2_-0.1 M HNO_3_ solution, nearly 15 times more than that in the 0.1 M HNO_3_ solution. The pseudo-second-order kinetic model and the Langmuir isotherm equation are suitable for expressing the adsorption characters of Ru(III) on SiPyR-N3, in which the adsorption process is endothermic. SiPyR-N3 has good adsorption ability and selectivity of about 100 for Ru in simulated HLLW with *SF*_Ru/other metal ions_, except for Zr, in 0.1 M NaNO_2_-0.1 M HNO_3_ solution. The adsorption mechanism involves the complexation of the pyridine functional group (C-N=C) with Ru(NO)^3+^ and its complexes with NO_2_^−^ and NO_3_^−^, as well as the anion exchange mechanism between protonated amine in SiPyR-N3 and Ru complex anionic species. Furthermore, Ru could be effectively desorbed by 0.1 M HNO_3_ + 1 M thiourea, and the desorption rate reached 92% at 328 K, which increased with increasing temperature.

## Data Availability

The data presented in this study are available on request from the corresponding author.

## Supplementary Materials

The following supporting information can be downloaded at: https://www.mdpi.com/article/10.3390/toxics12030181/s1, Table S1: Kinetic parameters of SiPyR-N3 adsorption of Ru in nitric acid system in nitric acid–nitrite system at 298, 308, and 318 K; Table S2: The adsorption isotherm parameters of two adsorption models for Ru on SiPyR-N3 in two systems at 298, 308, and 318 K; Table S3: Thermodynamic parameters of Ru adsorption on SiPyR-N3 in nitric acid system and in nitric acid–nitrite system; Figure S1: Effect of initial NaNO_2_ concentration on adsorption. (*m/V* = 0.02 g mL^−1^, *C*_Ru_ = 2 mmol L^−1^, *C*_HNO3_ = 0.1 mol L^−1^, *T* = 298 K); Figure S2: Effect of initial HNO_3_ concentration on adsorption. (*m/V* = 0.02 g mL^−1^, *C*_Ru_ = 2 mmol L^−1^, *T* = 298 K).
