# Supplementary material for: Adsorption and Desorption Behavior and Mechanism of Ruthenium in Nitrite–Nitric Acid System
Source: Toxics. 2024 Feb 27;12(3):181. doi: 10.3390/toxics12030181 (PMC10975168; doi:10.3390/toxics12030181)
Supplement: Supplementary file 1 [file toxics-12-00181-s001.zip › toxics-2870693-supplementary.pdf]

Table S1. Kinetic parameters of SiPyR-N3 adsorption of Ru in nitric acid system in nitric acid-nitrite system at 298, 308, 318 K

system	T (K)	Pseudo-first-order model			Pseudo-second-order model			$Q_{e,exp}$ (mg/g)
		K_1 (h^{-1})	Q_e (mg g^{-1})	R^2	K_2 (g mg^{-1} h^{-1})	Q_e (mg g^{-1})	R^2	
Nitric acid	298	4.49	1.53	0.556	1.03	1.91	0.999	1.89
	308	5.72	1.82	0.675	1.54	2.18	0.999	2.17
	318	1.95	2.06	0.676	1.06	2.47	0.999	2.45
Nitric acid sodium nitrite	298	4.03	8.43	0.642	6.83	8.58	0.999	8.57
	308	2.83	8.89	0.328	4.04	9.01	0.999	8.96
	318	2.49	9.09	0.913	8.55	9.21	0.999	9.18

Table S2. The adsorption isotherm parameters of two adsorption models for Ru on SiPyR-N3 in two systems at 298, 308, and 318 K

system	T (K)	Langmuir isotherm			Freundlich isotherm			$Q_{e,exp}$ (mg g^{-1})
		Q_m (mg g^{-1})	K_L (L mg^{-1})	R^2	N	K_F (mg^{1-n} L^n g^{-1})	R^2	
Nitric acid	318	11.99	6.89	0.944	1.59	0.31	0.901	11.94
	308	11.90	9.43	0.966	1.47	0.13	0.924	11.23
	298	9.42	2.36	0.964	1.43	0.11	0.917	9.88
Nitric acid sodium nitrite	318	45.70	0.02	0.984	4.39	7.59	0.867	45.57
	308	34.33	0.01	0.994	4.65	6.18	0.852	33.97
	298	30.86	0.01	0.998	4.76	5.33	0.874	30.57

Table S3. Thermodynamic parameters of Ru adsorption on SiPyR-N3 in nitric acid system in nitric acid-nitrite system

T（K）	Nitric acid			Nitric acid sodium nitrite		
	ΔH^o （kJ mol^{-1}）	ΔS^o J/(mol · K)	ΔG^o (kJ mol^{-1})	ΔH^o （kJ mol^{-1}）	ΔS^o J/(mol · K)	ΔG^o (kJ mol^{-1})
298	8.86	53.79	-7.15	21.24	117.89	-13.89
308			-7.71			-15.07
318			-8.25			-16.25
328			-8.78			-17.43

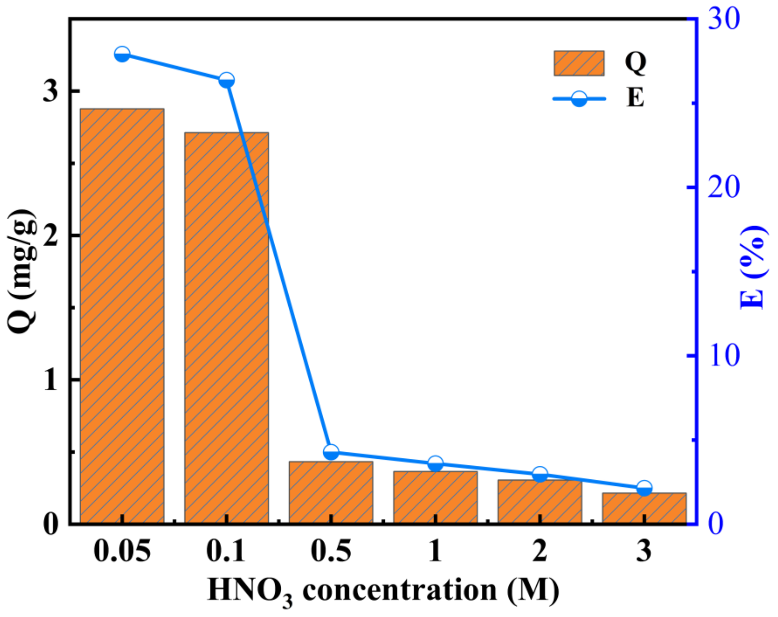

Figure S1. Effect of initial HNO_3 concentration on adsorption. (m/V = 0.02 g mL^{-1}, C_{Ru} = 2 mmol L^{-1}, T = 298 K).

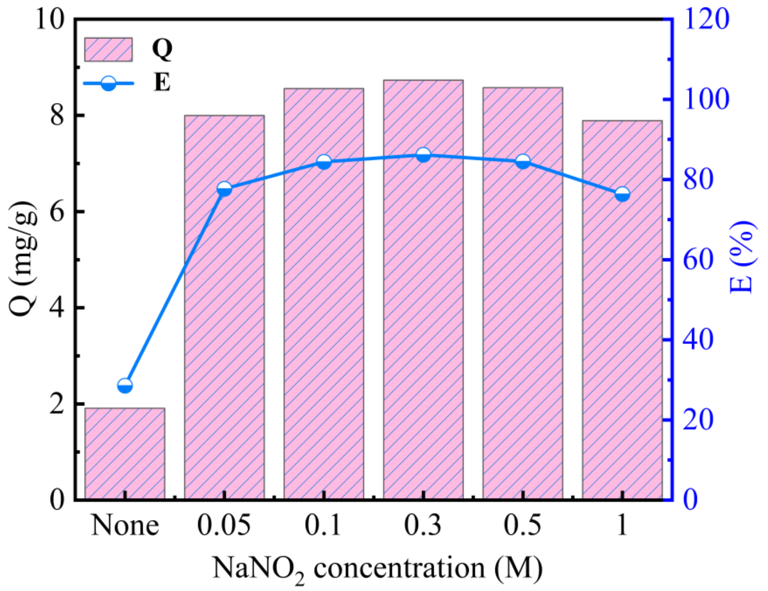

Figure S2. Effect of initial $NaNO_2$ concentration on adsorption. (m/V = 0.02 g mL^{-1}, C_{Ru} = 2 mmol L^{-1}, C_{HNO3} = 0.1 mol L^{-1}, T = 298 K).
